# The intestinal flora of patients with GHPA affects the growth and the expression of PD-L1 of tumor

**DOI:** 10.1007/s00262-021-03080-6

**Published:** 2021-10-13

**Authors:** Ding Nie, Qiuyue Fang, Jianhua Cheng, Bin Li, Mingxuan Li, Hongyun Wang, Chuzhong Li, Songbai Gui, Yazhuo Zhang, Peng Zhao

**Affiliations:** 1 Department of Neurosurgery, Beijing Tiantan Hospital, Capital Medical University, Beijing, China; 2 Beijing Neurosurgical Institute, Beijing, China

**Keywords:** Pituitary adenoma, Intestinal flora, Immune, PD-L1

## Abstract

**Context:**

Pituitary adenoma (PA) is a common intracranial tumor. The evidence indicates that the tumor immune microenvironment (TIME) is associated with PA and that the intestinal flora influences other tumors' growth through interacting with the TIME. However, how the intestinal microbial flora contributes to the development of PA through the immune response is unknown.

**Objective and methods:**

Here we used high-throughput Illumina MiSeq sequencing targeting the V3−V4 region of the 16S ribosomal RNA gene to investigate the intestinal flora of patients with growth hormone-secreting pituitary adenoma (GHPA), nonfunctional pituitary adenoma (NFPA), and healthy controls. We determined their effects on tumor growth and the TIME. Fecal microbiota transplantation (FMT) was performed after adoptive transfer via peripheral blood mononuclear cells to tumor-bearing nude mice, which allowed the study of the immune response.

**Result:**

We discovered differences in the structures and quantities of intestinal flora between patients with GHPA, patients with NFPA, and healthy controls. After FMT, the intestinal flora of GHPA patients promoted the growth of tumors in mouse models. The number of programmed cell death ligand 1 (PD-L1)-positive cells increased in tumor tissues as well as the extent of infiltration of CD8^+^ cells. Increased numbers of CD3^+^CD8^+^ cells and increased levels of sPD-L1 were detected in peripheral blood.

**Conclusion:**

These findings indicated that the intestinal flora of patients with GHPA promoted tumor growth and that the immune system may mediate this change.

**Supplementary Information:**

The online version contains supplementary material available at 10.1007/s00262-021-03080-6.

## Introduction

Pituitary adenoma (PA) is a common endocrine tumor of the central nervous system, which is classified according to hormone secretion as functional pituitary adenoma (FPA) and nonfunctional pituitary adenoma (NFPA) [1]. Growth hormone-secreting pituitary adenoma (GHPA) is a common subtype of FPA [2]. Excess production of GH leads to acromegaly, heart disease, sleep apnea, and other conditions that shorten life expectancy [3]. After systemic treatment (surgery and medication), certain GHPAs challenge effective clinical treatment because of relapse or drug resistance [4, 5]. Therefore, factors affecting the occurrence and development of GHPA must be identified.

Inflammatory immune cells, chemokines, and cytokines influence tumor growth and invasion [6]. Multiple studies show that GHPAs are associated with more CD4^+^ cells and CD8^+^ cells than non-GH adenomas as well as increased levels of programmed death ligand 1 (PD-L1) [7–10]. PD-L1 binds to the PD-1 receptor on activated *T* cells and inhibits the cytotoxic antitumor function of *T* cells [11–13]. For example, anti-PD-L1 treatment reduces plasma ACTH levels of model mice, decreases the growth of adenomas, and increases the survival rate of model mice [10]. Although no further study was conducted in that report, the expression of PD-L1 in GHPA was the highest in their study [10]. However, the factors affecting tumor expression of PD-L1 are unknown and must be identified to optimize immune checkpoint blockade therapy of PA.

The composition of the intestinal flora regulates the development of the central nervous system and contributes to the development of nervous system pathologies [14, 15]. The intestinal flora is a key environmental factor that affects the immune homeostasis of the host [16, 17]. For example, 11 distinct bacterial communities activate mouse CD8^+^ *T* cells to effectively inhibited tumor growth [18]. *Bifidobacterium* species enhance the antitumor effects of PD-L1 inhibitors as well as the functions of dendritic cells and CD8^+^ *T* cell-mediated antitumor mechanisms [19]. However, to our knowledge, direct evidence does not support the conclusion that the composition of intestinal microbiota affects the development or progression of PAs. Therefore, here we sequenced the bacterial flora in stool samples of healthy controls and patients with NFPA or GHPA. We determined the immune markers of GHPA and NFPA. We used immune reductionist mice to determine the effects of FMT on tumor formation by xenotransplanted GH3 cells. The changes in tumor and immune indexes were observed.

## Materials and methods

### Clinical studies and specimen processing

All studies were conducted under the approval of the Institutional Review Committee of Beijing Tiantan Hospital, Capital Medical University, and the study was conducted by the Principles of Good Clinical Practice and the Declaration of Helsinki. 50 patients with PA (25 GHPA,25 NFPA) were recruited from Beijing Tiantan Hospital, Capital Medical University. Twenty-five healthy subjects were recruited from a qualified population in Beijing, China.

Patients, who also met pathologically confirmed PA (according to the standards of The World Health Organization (WHO) classifications of tumors), had no history of chronic digestive tract disease, no other diseases (cold, etc.) on admission, had no history of chronic metabolic disease, did not take antibiotics or microbial agents within 1 week, had no history of cardiovascular disease, malignant tumor or other diseases, and had no history of smoking or alcohol abuse were considered to meet the inclusion criteria. Patients who did not meet the inclusion criteria were excluded. Controls were matched to PA patients concerning age, sex, following the same exclusion criteria as used for PA patients (Supplementary Table).

A total of 75 fresh feces were collected preoperatively, and all the donors fasted for 12 h before collection. Each sample was separately collected with three sterile PV tubes in the morning, sealed and temporarily stored in − 20℃, and transferred to − 80℃ within 2 h for long-term storage. Human blood samples were collected from the 75 subjects after overnight fasting. Serum samples were centrifuged and stored at − 80 °C.

### DNA extraction and 16SrRNA gene amplification

Bacterial DNA extraction and PCR amplification of the V3−V4 region of 16SrRNA gene was performed in Novogene Bioinformatics Technology Co., Ltd. The Illumina MiSeq platform was used to sequence the readings and obtain the results.

### Mice study

The contents and procedures related to animal testing involved in this study have been approved by the Institutional Animal Care and Use Committee (IACUC).

Female B-NDG(NOD-Prkdc^scid^ IL2rg^tm1^/Bcgen)mice were purchased from Biocyto (Beijing) Co., Ltd, with the age of 7–8 weeks and the weight of 200–220 g. In the absence of specific pathogens, all mice were raised in the barrier system of Serve Accurate Faithful Evaluation Co. (temperature, 20–26 ℃; humidity, 40–70%; 12-h light/12-h dark cycle) and fed a rodent diet ad libitum.

The cell line was Wistar Furth rat GH3 cells purchased from ATCC. The cell suspension with a concentration of 2.5 × 10^7^ cells/mL was subcutaneously injected into the back of mice near the armpit. Each mouse was inoculated with 0.2 mL and the inoculation dose was 5 × 10^6^ cells/ mouse.

### Immune reductionist

Female Wistar rats were purchased from Charles River (Beijing), with the age of 7–8 weeks, and were raised in the same environment as mice.

After the rats were anesthetized with isoflurane, the abdominal cavity was opened, and all blood (8–15 mL) of the rats was collected through the abdominal aorta in the heparin anticoagulant tube until the rats died. PBMC of Wistar rat was extracted by gradient centrifugation using the rat peripheral blood monocyte isolation liquid kit. The PBMC was injected into B-NDG tumor-bearing mice by tail vein injection, 0.5–1 × 10^7^ cells per mice. After 3 days, 0.2–0.3 mL of peripheral blood was collected from an orbital vein in an anticoagulant tube. CD45^+^ and CD3^+^ cells in the peripheral blood of mice were determined by flow cytometry to verify the results of immune remodeling. When CD45^+^ cells can be detected in the peripheral blood of mice, the immune reductionist is considered to be successful, and CD3^+^ cells confirmed the presence of *T* cells [20–22].

### Fecal microbiota transplantation

Mice with successful immune reductionists were placed in autoclaved drinking water with a mixture of broad spectrum antibiotics (1 g/L ampicillin,1 g/L metronidazole,1 g/L neomycin, and 0.5 g/L vancomycin) in the middle. Fresh stool samples were collected from two NFPA patients, two GHPA patients, and two control patients. The 500 mg of fecal samples was dissolved in 5 mL sterile saline, shaken well for 3 min, and centrifuged at 4 ℃ for 3 min, and the supernatant was collected and used as a transplant material. To reduce changes in the microbial population, fresh supernatants were prepared within 30 min prior to use. After 1 week of antibiotic treatment, the mice were given 150 μL of the above supernatant by gavage twice at a 1-day interval [23].

### Tissue processing

The mice were euthanized by CO_2_ asphyxiation, and the venous blood was collected. The cells of CD45^+^, CD3^+^CD4^+^, and CD3^+^CD8^+^ were measured by the Accuri^tm^ C6 Plus flow cytometer. After centrifugation, serum samples were obtained, and the content of sPD-L1 was measured by ELISA kits. The subcutaneous tumor was removed, and the long and short diameters of the tumor were measured with vernier calipers. Tumor volume was calculated by a vernier caliper. An electronic balance was used to weigh the tumor. Colonic contents were retained for 16SrRNA sequencing.

### Immunohistochemistry

Fifty surgical specimens and the tumors of mice were fixed with formalin, treated with routine treatment, fixed with paraffin, and cut into 4 μm sections for IHC. We used primary antibodies against PD-L1 (Bioss), CD8 (ab237709), and CD4 (ab133616, Bioss). A pathologist examined all the images. The number of CD4^+^ and CD8^+^ cells is expressed as an average of five random high-power fields (HPF). Intravascular positive cells were not counted. PD-L1 positive staining was defined as > 1% cellular reactivity.

### ELISA

Serum specimens are stored in a refrigerator at − 80 °C. According to the instructions of the ELISA kit(FANKEW), the optical density value of the final reaction product on the microplate analyzer was plotted at 450 nm. Concentrations were calculated from absorbance readings on CD4, CD8, and sPD-L1 standard curves.

### Flow cytometry

Antibody purchased from MULTISCIENCES: (CD45:70-AR4505; CD3:70-AR00301; CD4:70-AR0040210; CD8:70-AR00804). Blood samples were divided into two groups and tested under the same conditions, one group for CD45, CD3, and CD8, and the other group for CD3 and CD4. Appropriate isotype controls were used when applicable. The Accuri^tm^ C6 Plus flow cytometer was used for detection.

### Statistical analysis

The data analysis was performed using Prism 8.0 software (GraphPad), and analyzed by one-way ANOVA with Tukey’s multiple comparisons test, or by paired or unpaired student’s *t* test as indicated. Data were considered to be significantly different at *P* < 0.05, *P* < 0.01, and *P* < 0.001 represented in the figures and tables as *, **, and ***, respectively. Figures were processed using GraphPad Prism 8, Adobe illustrator CS5, and Adobe Photoshop CS4 software.

## Results

### Characteristics of the intestinal flora

**Fig. 1:**
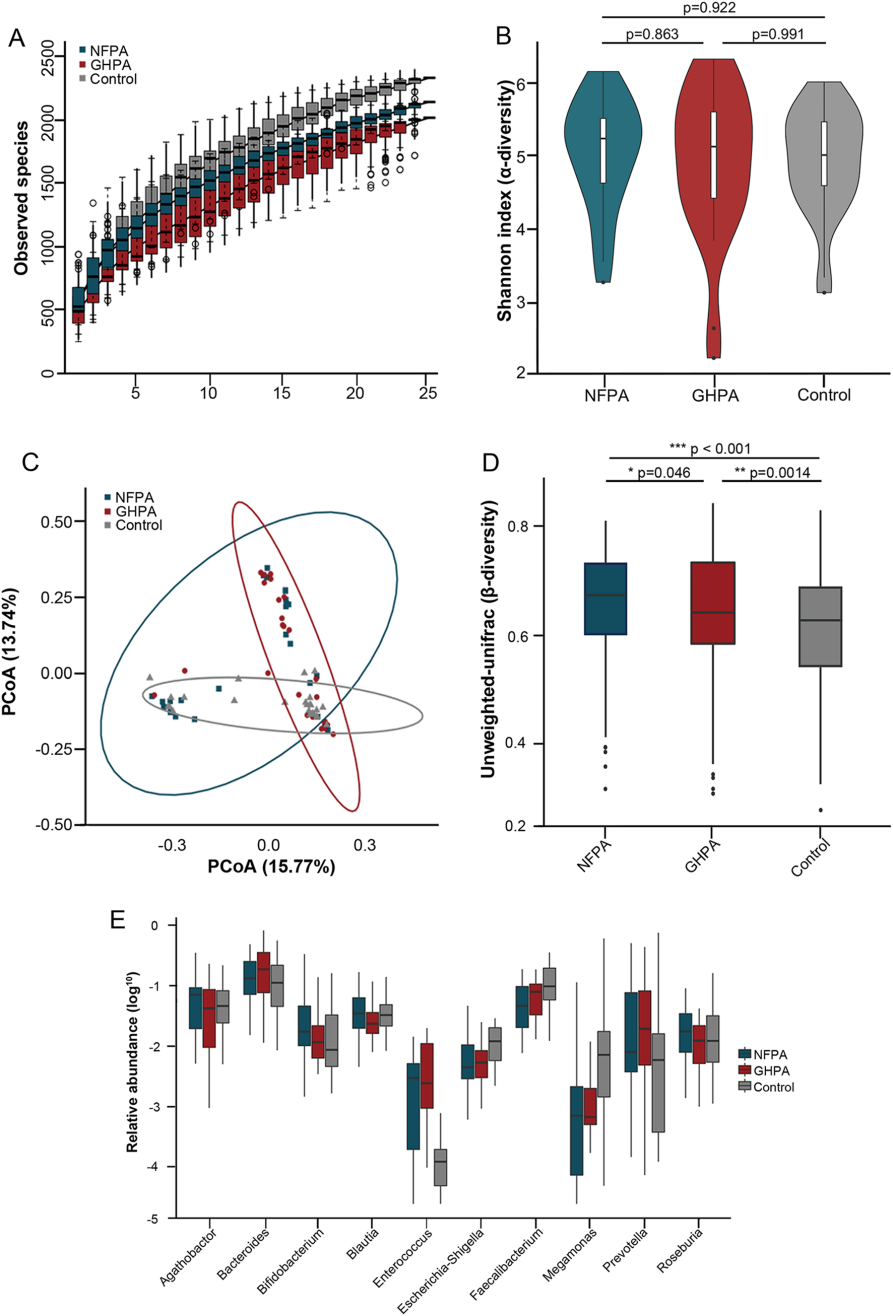
Characteristics of intestinal flora of the GHPA, NFPA, and healthy control groups. **a** Intestinal flora species accumulation of the GHPA (*n* = 25), NFPA (*n* = 25), and healthy groups (*n* = 25). **b** Fecal microbiome. The *α*-diversities of fecal microbiomes were evaluated using Shannon index according to the number of OTUs. **c** Principal coordinate analysis of samples from the GHPA, NFPA, and control groups. Each symbol represents a sample. **d** The *β*-diversities among the three groups significantly differed among groups. **e** Top 10 genera with the greatest differences in relative abundance. Each box plot depicts the values of the median, dispersion, maximum, minimum, and outliers

To study the intestinal flora of patients with different types of PAs and those of healthy controls, fecal samples of 75 donors from three groups were analyzed using high-throughput sequencing targeting the V3−V4 region of the 16S rRNA gene. As the sample size of each group increased, we found that species diversity increased and eventually plateaued, indicating that there was sufficient sampling for data analysis (Fig. 1a). The Shannon index indicated no significant difference in *α*-diversity among the three groups (Fig. 1b). Thus, there was no significant difference in species richness and evenness of intestinal flora. We further investigated the differences in species between samples. Ordination of unweighted-UniFrac dissimilarity using principal coordinate analysis (PCoA) revealed separation of the three groups (Fig. 1c). The significant differences in *β*-diversity among the three groups of intestinal flora indicate that separation was associated with differences among community compositions (Fig. 1d). Furthermore, at the genus level, we observed differences in the intestinal flora between the three groups. *Bacteroides*, *Biautia*, *Enterococcus*, *Megamonas,* as well as other genera were differentially represented among the three groups (Fig. 1e). There generally were significant differences among the compositions of the intestinal flora of patients with NFPA, GHPA, and those of healthy controls.

### Prediction of the function of the intestinal flora

**Fig. 2:**
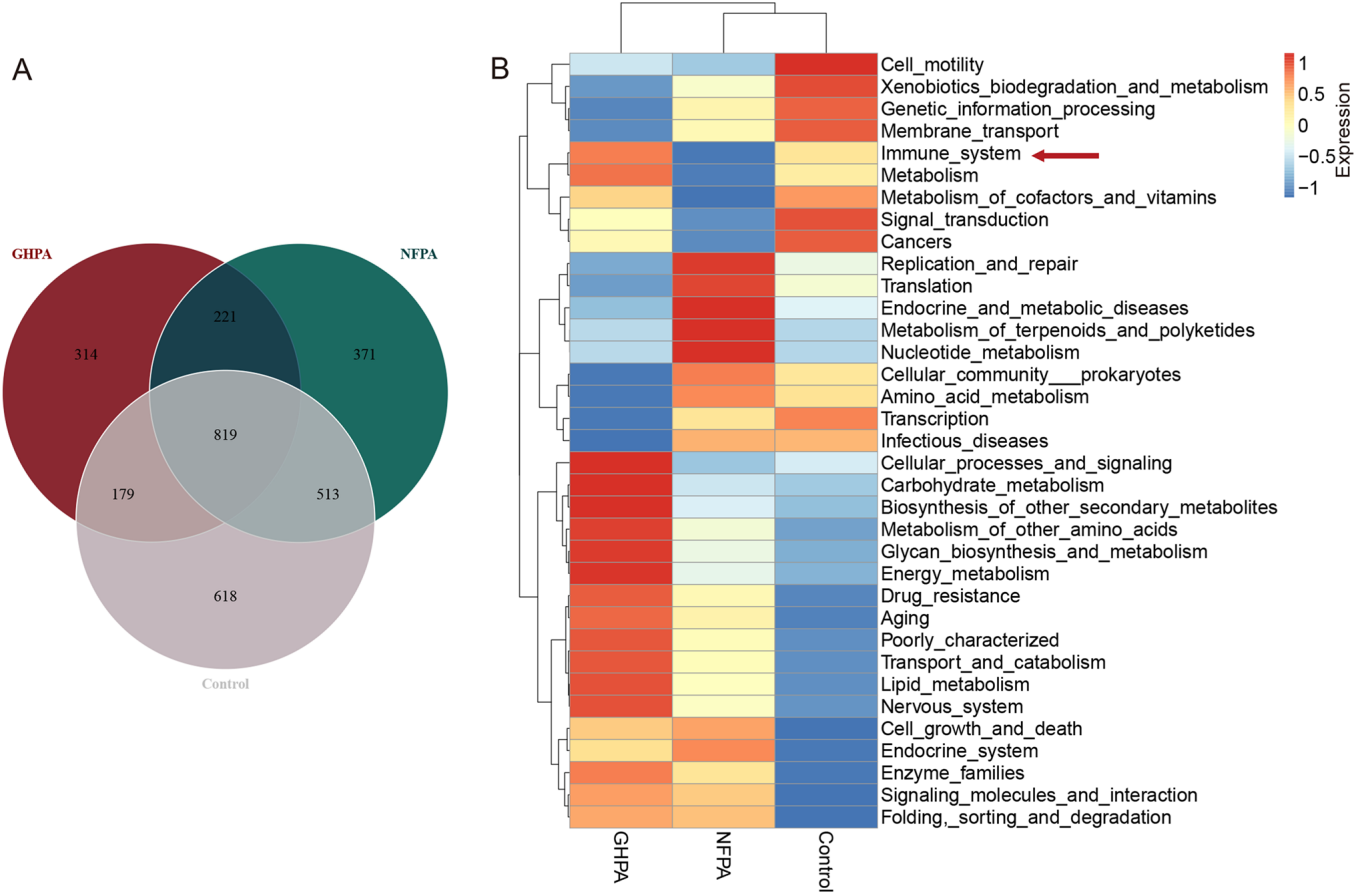
Prediction of intestinal flora function. **a** Unique numbers of OTUs of the three groups. (**b**) Functional annotation of samples and heat-map clustering of abundance information

We next evaluated species-level functions. For this purpose, we analyzed operational taxonomic units (OTUs) by generating a Venn diagram according to clustering. The numbers of OTUs specific to the GHPA, NFPA, and control groups were 314, 371, and 618, respectively (Fig. 2a). Functional analysis of intestinal flora in different groups indicated that the intestinal microflora in the three groups differentially affected the immune system (Fig. 2b). Overall, the influence of immune systems of the three groups of flora exhibited functional differences.

### Immune characteristics of PA

**Fig. 3:**
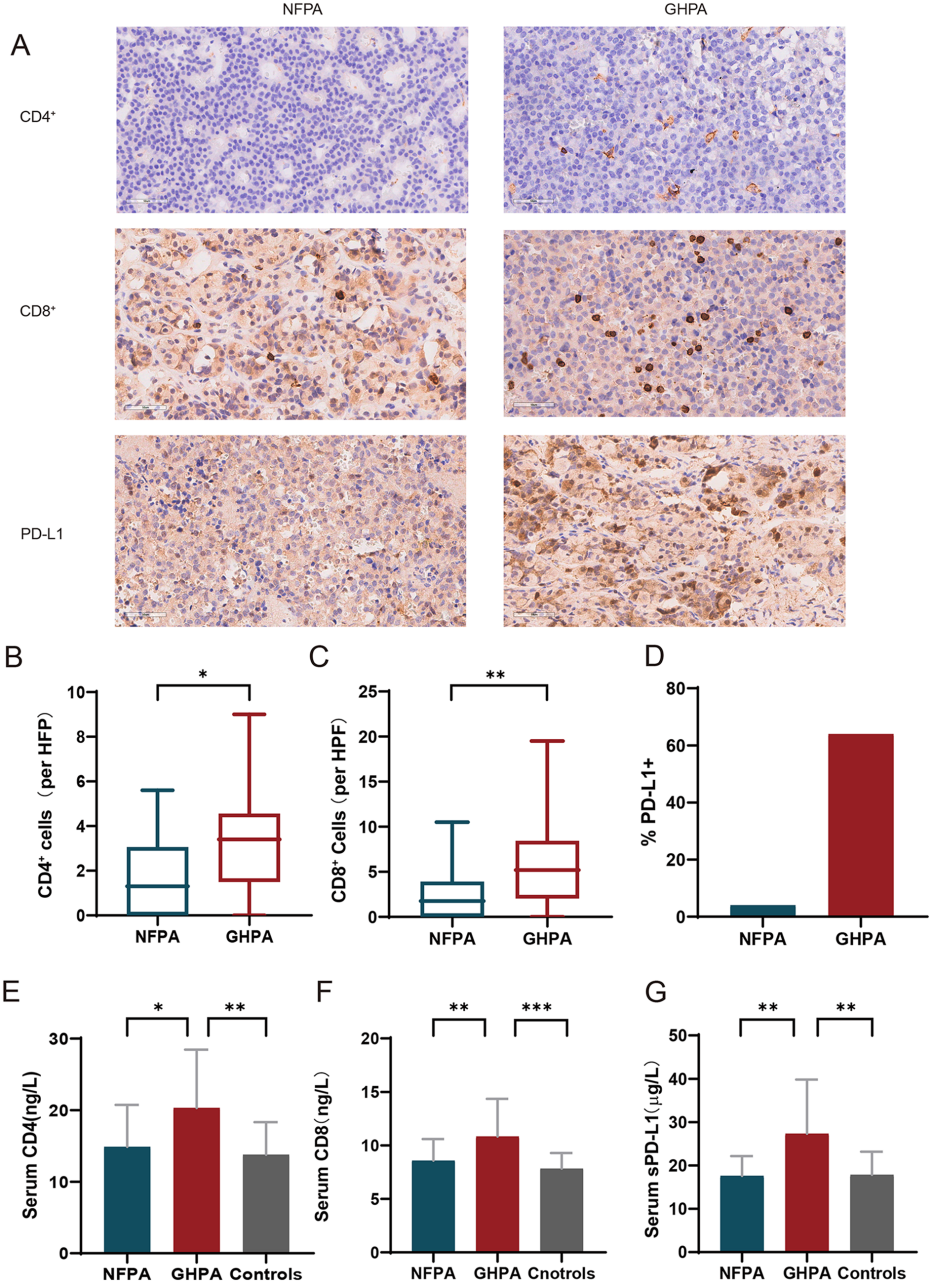
IHC analysis of the expression of PD-L1 and the numbers of CD4^+^ cells and CD8^+^ cells in the GHPA (*n* = 25) and NFPA (*n* = 25) groups; and serum ELISA results. **a** Representative IHC data of GHPA and NFPA samples showing that PD-L1 levels and numbers of CD8^+^ cells and CD4^+^ cells were frequently higher in GHPA samples. PD-L1 positivity was defined as > 1% staining of cells. **b**, **c** Numbers of CD4^+^ cells in the GHPA group versus the NFPA group (*P* = 0.0197). Numbers of CD8^+^ cells in the GHPA and NFPA groups (*P* = 0.0086). **d** PD-L1-positivity rates in the GHPA group vs the NFPA group (64% vs. 4%). **e**–**g** The serum levels of CD4, CD8 and sPD-L1 in patients with GHPA were significantly higher compared with those of the other two groups

To evaluate the activities of immune cells and PD-L1 expression in GHPA compared with NFPA, we simultaneously analyzed tumor and peripheral blood samples. Examples of IHC analysis of the numbers of CD4^+^ cells and CD8^+^ cells and PD-L1 expression are shown in Fig. 3a. CD4^+^ cells and CD8^+^ cells were more frequent in GHPA than in NFPA samples (Fig. 3b, c), and the proportions of PD-L1-positive tumors in GHPA and NFPA samples were 64% (16/25) and 4% (1/25), respectively (Fig. 3d). When we measured the levels of the three markers in serum samples from the corresponding patients and healthy controls, we found that the levels of CD4, CD8, and sPD-L1 in patients with GHPA were significantly higher compared with those in NFPA patients and healthy controls and that there was no significant difference between patients with NFPA and healthy controls (Fig. 3e–g). These data indicate that patients with GHPA had a distinct immune environment, particularly that associated with the TIME.

### GHPA-FMT promotes tumor growth

**Fig. 4:**
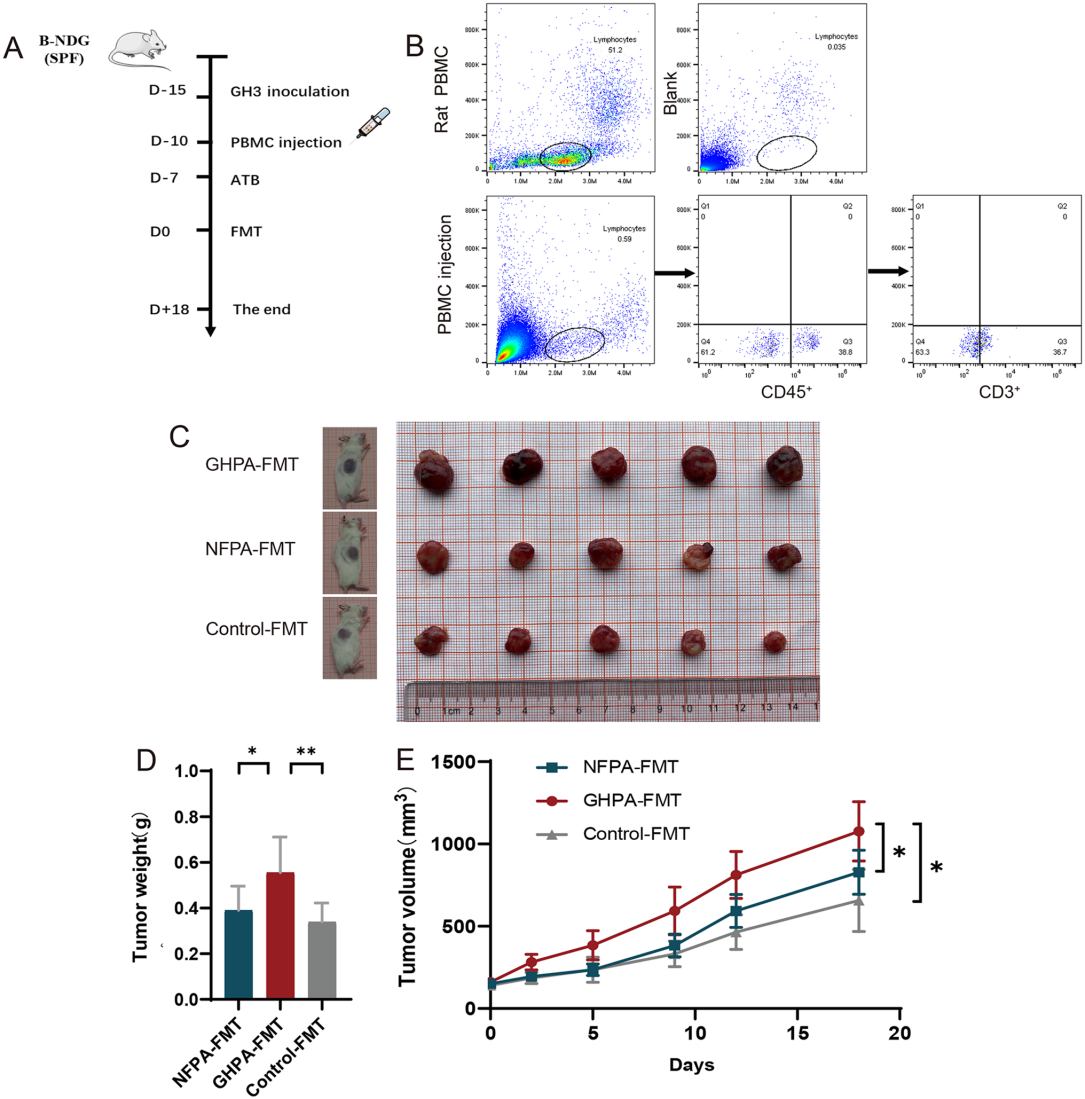
GHPA-FMT promotes tumor growth. **a** Experimental scheme. **b** Representative flow cytometry analysis. After adoptive transfer of PBMCs, lymphocytes, CD45^+^ cells, and CD3^+^ cells were detected in the peripheral blood of mice. There were no lymphocytes in the control group. **c**–**e** Images of the solid tumor (**c**), tumor weights (**d**), and tumor volumes (**e**)

To determine the influence of the intestinal flora on the growth of tumors and immune functions, we established a subcutaneous tumor model of mice with immunodeficiency and injected PBMC extracted from the blood of Wistar rats through the tail vein to achieve immune reconstruction. The mice were then given broad-spectrum antibiotic (ATB) compounds for 1 week to deplete intestinal flora. Finally, 30 mice were divided into three groups with 10 mice in each group. The mice in the three groups were, respectively, gavaged with the fecal supernatant of two NFPA patients, two GHPA patients, and two control donors to realize FMT (Fig. 4a). The presence of T lymphocytes in peripheral blood was detected using flow cytometry after mice received reinfusion of PMBCs (Fig. 4b). To prove whether FMT regulated the intestinal microflora of the mice, we sequenced the 16S rRNA (V3 + V4 regions) of the colon contents and found that the composition of the intestinal microflora of the three groups of mice differed and showed similar results in functional prediction as in human samples (Supplementary Fig. 1). The tumor phenotype is shown in (Fig. 4c). GHPA-FMT significantly increased tumor weight and volume compared with the Control-FMT and NFPA-FMT groups (Fig. 4d, e). These data indicate that the growth of tumors in GHPA-FMT mice exceeded that of NFPA-FMT and Control-FMT mice.

### GHPA-FMT affects the systemic immune response

**Fig. 5:**
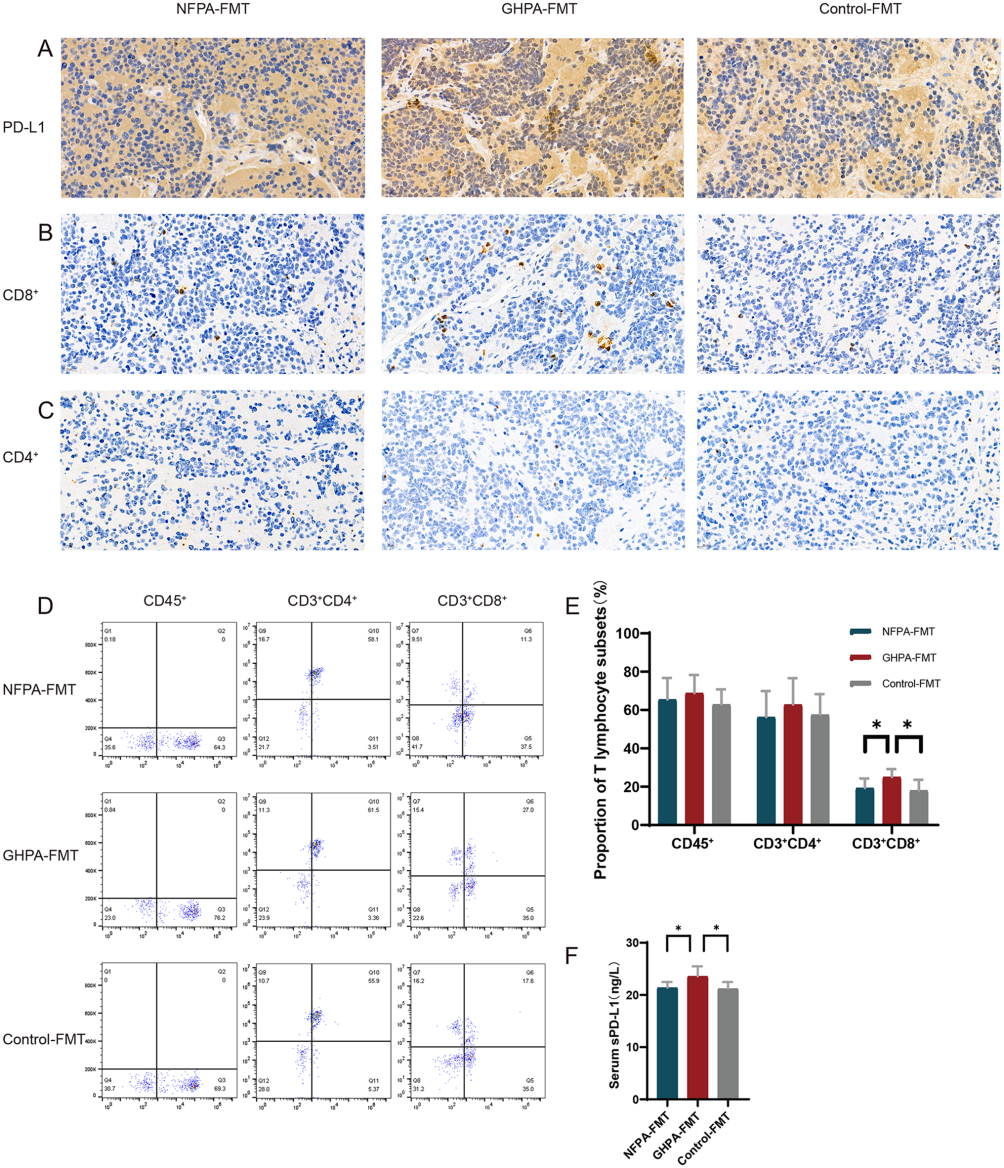
GHPA-FMT promotes the expression of PD-L1 and the infiltration of CD8^+^ cells in PAs and affects their numbers in peripheral blood. **a**–**c** Representative IHC analysis. Compared with NFPA-FMT and control-FMT groups, the GHPA-FMT group comprised significantly increased the numbers of PD-L1-positive cells and infiltration of CD8^+^ cells, although there was no significant difference in the number of CD4^+^ cells. **d**, **e** Representative flow cytometry analysis. GHPA-FMT significantly increased the proportion of CD3^+^CD8^+^ cells in peripheral blood. **f** Serum ELISA. GHPA-FMT significantly increased the levels of sPD-L1

To determine the influence of FMT on the immune system, we measured the levels of immune markers in tumors and peripheral blood. Representative IHC data for the expression of CD4, CD8, and PD-L1 are shown in Fig. 5a–c. In tumor tissues, GHPA-FMT increased the diffusely positive rate of PD-L1^+^ cells, and the number of PD-L1-positive cells was higher compared with those of the other two groups, which exhibited few PD-L1-positive cells (Fig. 5a). Furthermore, FMT promoted the infiltration of tumors by CD8^+^ cells, but not that of CD4^+^ cells (Fig. 5b, c). When we measured the proportion of T lymphocyte subsets in the blood (Fig. 5d), we found that GHPA-FMT increased the proportion of CD3^+^CD8^+^ cells in the blood (Fig. 5e). ELISAs revealed that GHPA-FMT increased the levels of sPD-L1 in peripheral blood (Fig. 5f). These results indicate that GHPA-FMT affected the changes of immune indexes in blood and the infiltration of tumors by immune cells.

## Discussion

**Fig. 6:**
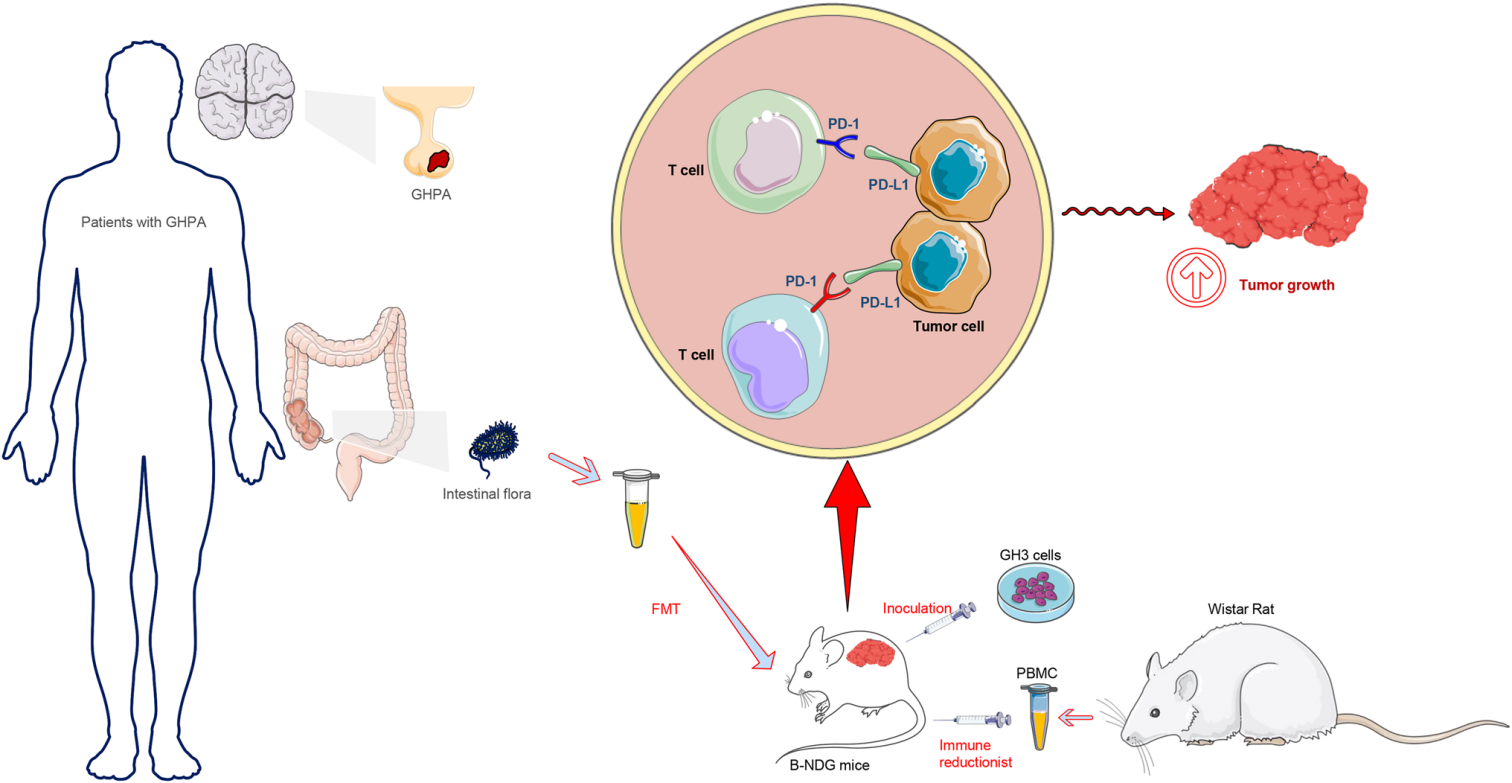
Experimental protocol. In B-NDG mice after immune reduction and tumor inoculation, PD-L1 levels in tumor tissues increased after GHPA-FMT, and tumor growth subsequently increased

The richness and diversity of the types of intestinal flora can reflect a person's health [24]. For example, intestinal flora is an important factor in central nervous system disease, contributing to the maintenance of the host’s immune homeostasis [14, 15]. However, there is no direct evidence of a relationship between intestinal flora and PAs. Here we identified significant differences in intestinal microbiota between patients with NFPA and those with GHPA, as well as healthy controls, and evaluated their effects on tumors using a mouse tumor xenograft model. Furthermore, to identify potential targets of the intestinal flora, we modified a mouse tumor model through immune reduction technology and found that after GHPA-FMT, the number of PD-L1-positive cells in tumor tissues increased, accompanied by increased infiltration of tumors by CD8^+^ cells, which was also reflected by the cognate lymphocyte subset in peripheral blood. Considering the complex relationship between PD-1 and PD-L1, we also analyzed the expression of PD-1 in tumor tissues, while no significant difference was observed (Supplementary Fig. 2).Our findings reveal potential mechanisms through which the intestinal flora affects PA (Fig. 6).

PD-L1 is expressed by numerous cell types, and its expression on the surface of tumor cells is a driving factor for tumor growth as the tumor escapes from the pursuit of immune cells [25]. The binding of PD-1 to PD-L1 alters the activity of *T* cells through numerous mechanisms, thereby inhibiting *T* cell proliferation, survival, and their ensuing effects [25]. Moreover, the level of infiltration of T cells in GHPA and the expression of PD-L1 are higher compared with other PAs, which is consistent with the results of our present study [8, 26–28]. We show here that the populations of CD4^+^ cells, CD8^+^ cells, and the levels of PD-L1 in GHPA were higher compared with those of NFPA, indicating a unique association of the TIME with GHPA. However, some reports indicate that the sizes of the populations of CD4^+^ cells did not significantly differ between GHPA and NFPA [29]. This may be explained by differences in sample selection and the relatively small sample size that must be increased in future research. The larger populations of CD4^+^ cells and CD8^+^ cells and increased serum levels of sPD-L1 in patients’ peripheral blood with GHPA suggest that the tumor not only affects the TIME but also profoundly exerts systemic effects involving immunity.

A stable microbial flora structure and human health are mutually reinforcing, and changes in one will lead to a corresponding response in the other. For the intestinal flora, the host influences the adaptability of individual bacteria in a competitive environment, and therefore the suboptimal function of the microbiota degrades the health of the host [30]. Here we found no significant difference in the α-diversity index (richness and diversity) of intestinal flora between the NFPA, GHPA, and control groups. However, there were significant differences in the *β*-diversity index (both structure and quantity) between the three groups, indicating that the composition of the intestinal microbiota of each significantly differed. This difference might be caused by a variety of factors, including diet, age, sex, hormone levels, and disease status [31]. GH and insulin-like growth factor 1 (IGF-1) in patients with GHPA are significantly higher than those with NFPA and normal controls [32]. Recently, researchers have found that the gut microbiota of male mice changes in response to excessive levels of GH [33]. In addition, gut microbiota can influence host hormone levels, such as auxin-releasing peptide and cortisol, suggesting a complex interaction between the GH/IGF-1 axis and intestinal flora [34, 35]. However, there were also differences in the composition of gut microbiota between the NFPA group and normal control, while no differences in hormone levels between the two groups. Thus, we speculate that the reasons for the differences in intestinal flora composition among the three groups in this study are complex and diverse, not limited to the hormone levels, which needs further study.

Robust data indicate that the composition of the intestinal flora is a major environmental factor that varies between individuals and influences systemic immunity [36]. For example, tumors in mice that receive intestinal flora from patients with metastatic melanoma sensitive to anti-PD-1 therapy have higher CD8^+^ *T* cell densities and higher levels of PD-L1 compared with those of the nonsensitive group [37]. Furthermore, antibiotic treatment of altered intestinal microflora in mice with gliomas changes the distribution of NK cell subsets and decreases antitumor effects, suggesting that the alteration of the intestinal flora may affect the growth of brain tumors by affecting the immune response [38]. Here we found that the number of PD-L1-positive cells in the tumors of GHPA-FMT mice was significantly higher compared with the NFPA-FMT and Control-FMT groups, which may represent an important factor leading to increased tumor growth in GHPA-FMT mice. Thus, GHPA-FMT caused an increase in immune escape from tumors. The factors affecting PD-L1 expression in tumors seem to be quite complex, including epigenetic changes, signaling pathways, and transcriptional regulation, cytokines and oxidative stress, etc. [39–41]. Robust data reveal that gut microbes can influence these factors. *Bacteroides* (one of the different species found in this study), for example, can convert cellulose, lignin, and pectin into short-chain fatty acids and subsequently affect the disease of the central nervous system through oxidative stress [42]. Intestinal flora can regulate IFN-γ production, and Freeman et al. demonstrated that IFNγ upregulates PD-L1 in monocytes and dendritic cells [43, 44]. We thus hypothesize that the gut microbiota could regulate PD-L1 expression of pituitary adenoma via regulation of metabolism and release of cytokines, and we will validate the hypothesis in our future work. Interestingly, a recent study indicated that the infiltration of CD8^ +^ T cells in the tumor was significantly positively correlated with the abundance of *Faecalibacterium*, *Ruminococcaceae*, and *Clostridiales* and negatively correlated with *Bacteroidales*(though not significant) [45], revealing intestinal flora may influence *T* cells infiltrating in tumors. Moreover, in colorectal cancer, intestinal microbiome-derived stimulation induces chemokine expression in tumor cells, ultimately enhancing *T* cell recruitment into tumor [46]. Our results showed more infiltration of CD8^+^ cells and higher PD-L1 expression in GHPA-FMT mice compared to the NFPA-FMT and control-FMT mice, while larger tumors were observed in the GHPA-FMT mice. We believe the limited increase in CD8^+^ cells in the GHPA-FMT mice may be explained not only by the changes in intestinal flora but also by compensation for immune escape after the increased PD-L1expression.When the function of immune cells is suppressed, the body’s immune system increases their numbers. Further research is required to prove this hypothesis. Moreover, there is evidence that the presence of CD8^+^ cells in tumors predicts the clinical response to anti-PD-1 therapy, and the increase in these cells after GHPA-FMT provides a basis for further exploration of GHPA-specific immunotherapy. In contrast, a similar result has not obtained for NFPA-FMT and Control-FMT mice, suggesting that differences in the intestinal flora between patients with GHPA and these latter groups may cause changes in the tumor microenvironment, thereby affecting tumor growth. Moreover, the increased proportion of CD3^+^CD8^+^ cells in peripheral blood and the elevated levels of sPD-L1 suggest that GHPA-FMT systemically affects the immune system.

There are several limitations to our study. First, we studied a mouse model of PA with the technical limitations of direct cellular implantation, which does not perfectly reproduce the anatomy of PA, although we believe we convincingly demonstrate the influence of intestinal flora on PA in vivo. Furthermore, the mouse model is relatively mature and stable as established by related studies of PA; and we provide proof of concept of the influence of intestinal flora on PA. Second, we used immune reconstruction technology to simulate the immune environment in animals, which is insufficient for comprehensive immune research. Moreover, highly complex factors affect tumor growth, immunity is only one of them. Finally, the mechanism through which intestinal flora influences tumor growth through immune response remains to be further explored, requiring a larger sample size and cell-level studies, which are our future research directions.

## Conclusion

The different compositions and distributions of the intestinal flora of patients with PA exert systemic effects on the immune system. GHPA-FMT promotes the growth of PA, which may be achieved through increasing the number of PD-L1-positive cells in tumors, thereby enhancing the escape of tumor cells from the immune response. There may be a compensatory increase in the infiltration rate of CD8^ +^ cells when their cytotoxic function is inhibited.

### Supplementary Information

Below is the link to the electronic supplementary material.

Supplementary Figure 1. Characteristics of intestinal flora in the of GHPA-FMT, NFPA-FMT, and control-FMT groups. (A) β-diversity. (B) Venn diagram of OUTs. (C) The flora differed among the three groups at the genus level. (D) Functional annotation of samples and heat-map clustering of abundance (TIFF 20730 KB)

Supplementary Figure 2. Expression of PD-1 in tumor tissue. (A) Representative IHC analysis of GHPA and NFPA samples. (B) Representative IHC analysis of GHPA-FMT, NFPA-FMT, and control-FMT groups (TIF 25509 KB)

Supplementary file3 (DOCX 17 KB)

## Data Availability

Some or all datasets generated during and analyzed during the current study are not publicly available but are available from the corresponding author on reasonable request.
