# Supplementary figures and images for: The intestinal flora of patients with GHPA affects the growth and the expression of PD-L1 of tumor
Source: Cancer Immunol Immunother. 2021 Oct 13;71(5):1233–45. doi: 10.1007/s00262-021-03080-6 (PMC9016060; doi:10.1007/s00262-021-03080-6)

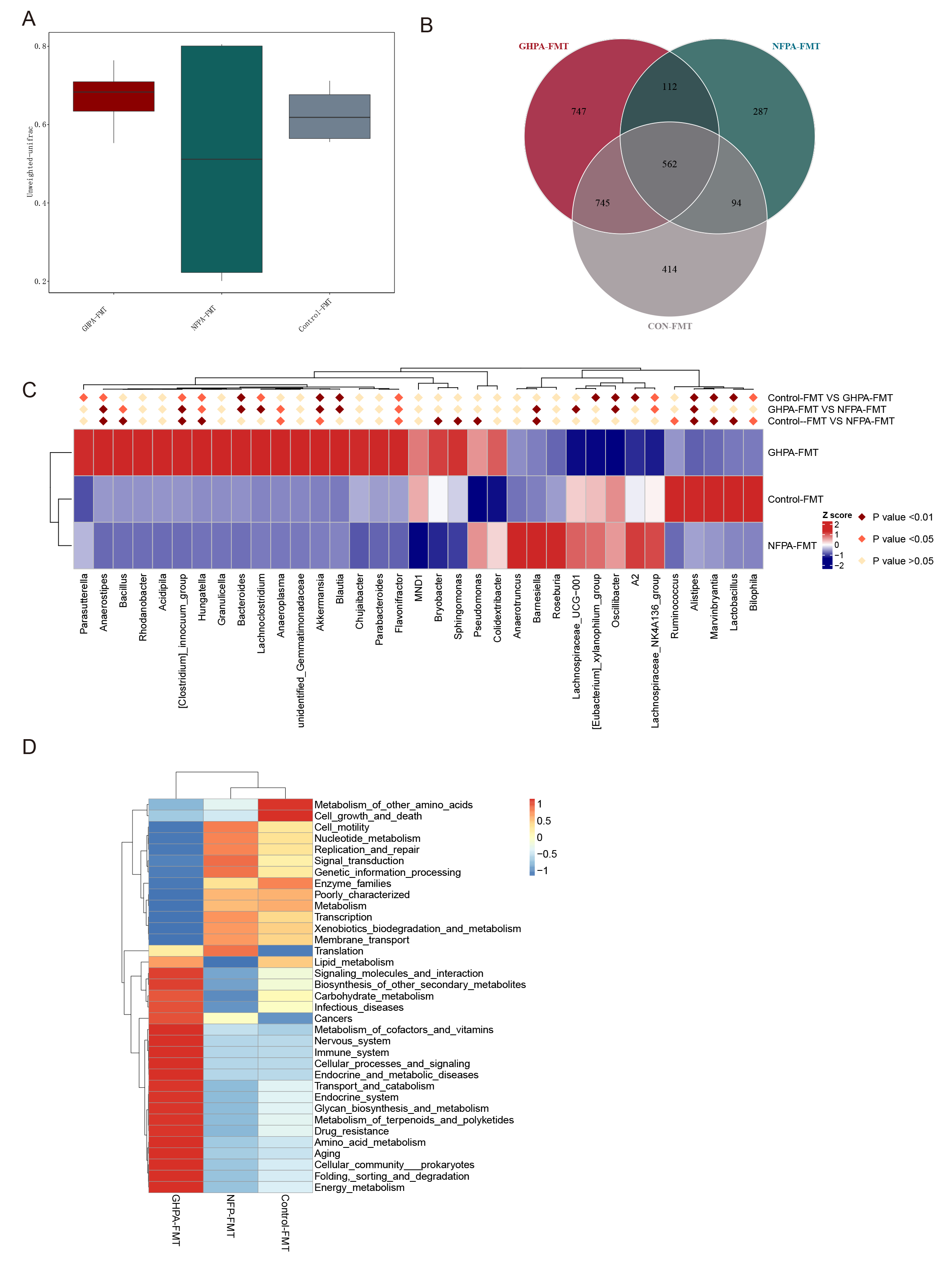

Supplement: Supplementary file 1 — Supplementary Figure 1. Characteristics of intestinal flora in the of GHPA-FMT, NFPA-FMT, and control-FMT groups. (A) β-diversity. (B) Venn diagram of OUTs. (C) The flora differed among the three groups at the genus level. (D) Functional annotation of samples and heat-map clustering of abundance (TIFF 20730 KB) [file 262_2021_3080_MOESM1_ESM.tiff]

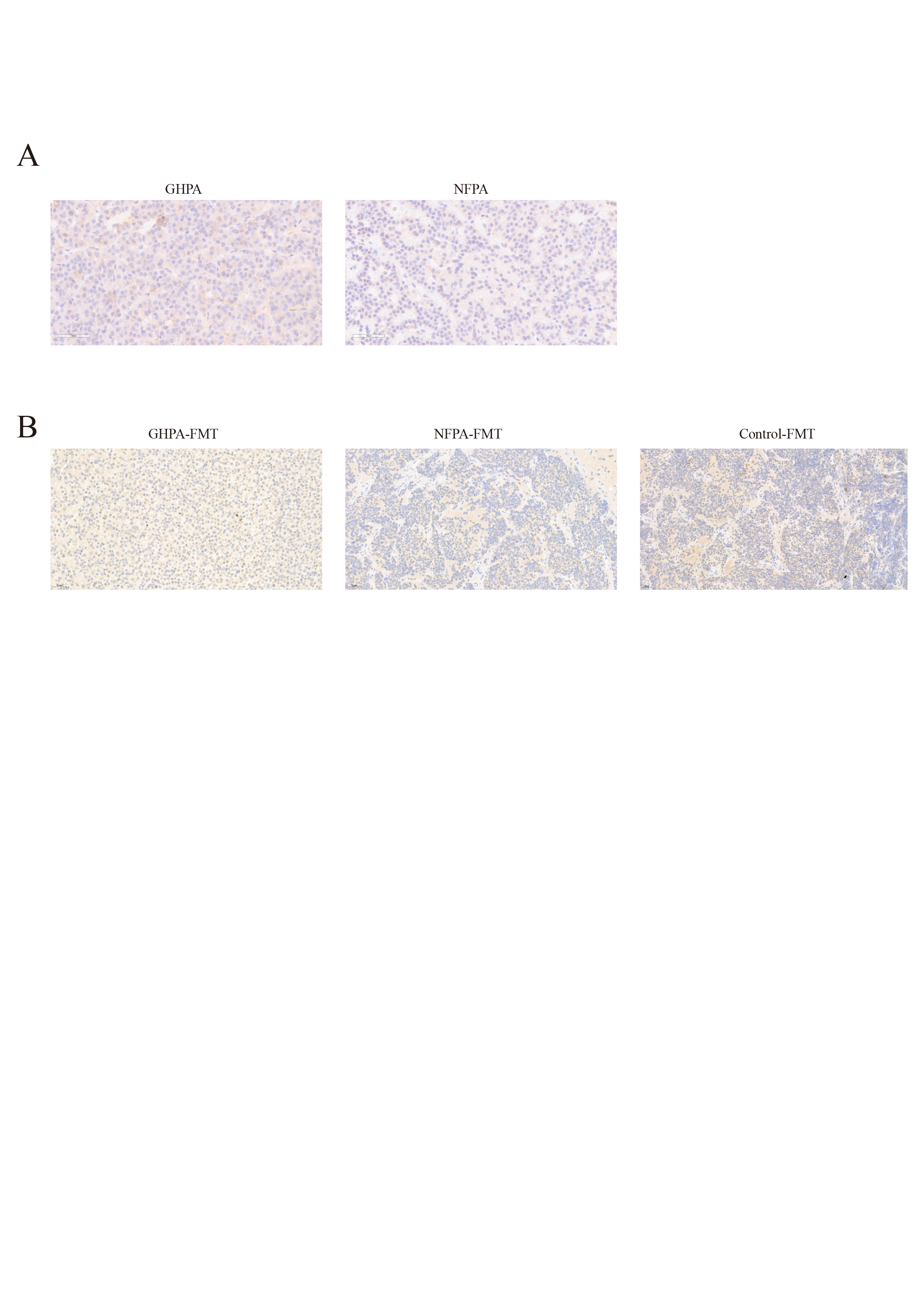

Supplement: Supplementary file 2 — Supplementary Figure 2. Expression of PD-1 in tumor tissue. (A) Representative IHC analysis of GHPA and NFPA samples. (B) Representative IHC analysis of GHPA-FMT, NFPA-FMT, and control-FMT groups (TIF 25509 KB) [file 262_2021_3080_MOESM2_ESM.tif]
