# Supplementary material for: The intestinal flora of patients with GHPA affects the growth and the expression of PD-L1 of tumor
Source: Cancer Immunol Immunother. 2021 Oct 13;71(5):1233–45. doi: 10.1007/s00262-021-03080-6 (PMC9016060; doi:10.1007/s00262-021-03080-6)
Supplement: Supplementary file 3 — Supplementary file3 (DOCX 17 KB) [file 262_2021_3080_MOESM3_ESM.docx]

| Characteristics | NFPA(n=25) | GHPA(n=25) | Controls(n=25) | Statistical significance |
| --- | --- | --- | --- | --- |
| Age, y | 47.08±6.87 | 46.28±7.05 | 45.25±7.58 | None |
| Male sex, % | 13 (52.0%) | 12(48.0%) | 13（52.0%） | None |
| BMI, kg/m^2^ | 29.12±6.98 | 32.97±8.09 | 30.50±4.21 | None |
| Medications | None | None | None | None |
| GH, ng/ml | 0.46±0.47 | 21.14±12.87 | 0.41±0.39 | GHPA vs. NFPA p<0.01  GHPA vs. Controls p<0.01  NFPA vs. Controls p>0.99 |
| IGF-1, ng/ml | 97.8±28.9 | 598.1±269.96 | 90.5±29.68 | GHPA vs. NFPA p<0.01  GHPA vs. Controls p<0.01  NFPA vs. Controls p>0.99 |

Supplementary Table. Clinical characteristics of 75 samples.

NFPA= nonfunctional pituitary adenoma; GHPA= growth hormone-secreting pituitary adenoma; BMI =body Mass Index. Normal range: GH (0-3.0ng/ml); IGF-1 (64-188ng/ml).
